# Compassion for others and well-being: a meta-analysis

**DOI:** 10.1038/s41598-025-23460-7

**Published:** 2025-10-20

**Authors:** M. Zhuniq, F. Winter, C. Aguilar-Raab

**Affiliations:** Clinical Psychology, Interaction- and Psychotherapy Research, Institute for Compassionate Awareness and Interdependence Research and Practice IN-CARE, School of Social Sciences, University of Mannheim, Mannheim, Germany

**Keywords:** Compassion for others, Empathic concern, Meta-analysis, Moderator, Systematic review, Well-being, Psychology, Quality of life

## Abstract

Compassion has been shown to improve well-being across multiple settings. While the link between self-compassion and well-being is well-established, findings on compassion for others have been more mixed. Using a multilevel approach, this meta-analysis of 54 effect sizes examined the relationship between compassion for others and well-being. The results revealed a moderate, statistically significant positive association (*r* = .26), suggesting that compassion for others contributes meaningfully to overall well-being. This relationship was consistent for psychological well-being, cognitive well-being, social well-being, and positive affect, whereas the results were weaker for negative affect. Furthermore, results showed that this relationship is not moderated by age, gender, or region. In addition, we examined if there is a causal relationship between compassion for others and well-being examining six effect sizes for state and trait compassion interventions. The results showed moderate improvements in well-being following compassion-based training, indicating promising but preliminary evidence for a causal effect. However, the absence of consistent control group comparisons limits firm conclusions about causality. This meta-analysis presents the first rigorous synthesis of evidence linking compassion for others to well-being, highlighting compassion for others as a distinct and meaningful contributor.

**Supplementary Information:**

The online version contains supplementary material available at 10.1038/s41598-025-23460-7.

## Introduction

Compassion is a multifaceted and sensitive response to suffering, encompassing affective, cognitive, and motivational components^1^. It involves the recognition of suffering, an understanding of its universality, empathy coupled with distress tolerance, and a motivational drive to alleviate distress^2^. Compassion can be directed toward others, toward oneself, or received from others. A growing body of research links compassion to improved health outcomes, as evidenced in both cross-sectional and intervention studies^3–5^. These benefits include reduced physiological stress reactivity^6–8^ and enhanced resilience in the face of stress^9,10^. Compassion is also associated with increased feelings of closeness toward socially disliked individuals and a reduction in schadenfreude^11^, greater social connectedness^12^, and elevated prosocial behavior^13^. Furthermore, compassion appears to support neuroplasticity^14,15^ and confers benefits across domains such as the workplace^16^, educational settings^17^, and clinical practice^18–20^.

Well-being, a central construct in health research, extends beyond the absence of psychopathology and encompasses various dimensions of individual functioning^21^. Cognitive and affective aspects of well-being originate in Bradburn’s^22^ affect balance theory, which posits that subjective well-being arises from the interaction of positive and negative affect, along with an evaluative judgment of life satisfaction. Psychological well-being, understood more broadly and multidimensionally, is grounded in Ryff’s^23^ model, which identifies six core components: self-acceptance, purpose in life, autonomy, personal growth, environmental mastery, and positive relations with others. While subjective and psychological well-being are related, they represent statistically distinct constructs^24^, reflecting two philosophical traditions: the hedonic (subjective well-being), which emphasizes pleasure and satisfaction^25^, and the eudaimonic (psychological well-being), which emphasizes meaning and self-realization^26^. The latter is frequently conceptualized through the lens of Self-Determination Theory, highlighting the roles of autonomy, competence, and relatedness.

Given humans’ inherently social nature, health is shaped not only by the degree of social integration (structural dimension) but also by the perceived quality and functionality of social relationships (functional and qualitative dimensions)^27^. Accordingly, social well-being is best understood as a distinct yet interrelated facet of overall well-being. The relevance of these dimensions is underscored by consistent meta-analytic evidence linking well-being to reduced mortality risk^28,29^, including robust associations specifically for social well-being across its structural, functional, and qualitative aspects^30–32^.

The association between self-compassion and well-being is well established. A large-scale meta-analysis found a moderate overall correlation, with stronger links to cognitive and psychological well-being than to affective well-being^33^. In contrast, compassion for others remains less thoroughly investigated, with prior findings yielding mixed results. Some studies suggest a positive association, particularly among individuals engaged in personal development (e.g., Buddhist practitioners or counseling students), compared to general population samples^34–36^. Additionally, values such as authenticity appear to strengthen this association^37^. However, other studies report only small or non-significant correlations in non-specialized samples, suggesting that self-compassion and compassion for others may be largely distinct constructs^38,39^. Considering these mixed findings, conclusions about self-compassion cannot be readily extended to compassion for others. Prior work suggests that demographic factors may moderate the effects on compassion; for instance^33^, shows that the proportion of females in samples strengthens the link between self-compassion and well-being, with marginal trends for age and region, where older participants and European samples show higher correlations between self-compassion and well-being compared to younger participants and North American samples. These findings provide a rationale to explore these moderators in the context of compassion for others as well. Research questions:

1. How does compassion for others relate to different forms of well-being?
2. Does age, gender, or region moderate this relationship?
3. Is there a causal relationship between compassion for others and well-being?

## Methods

### Eligibility

This meta-analysis is preregistered in PROSPERO (CRD42024538869) and follows the PRISMA guidelines^40^. Studies were included if they met the following eligibility criteria: a) quantitative studies that report a correlation between compassion for others (or empathic concern) and at least one form of well-being, b) compassion for others and well-being assessed using validated psychometric instruments, c) adult participants (aged 18 or older). Studies had to be in English, German, or Albanian, and no restriction based on publication date was placed. For compassion for others, we included empathic concern, as they are considered interchangeable in the literature^41^. We defined well-being in five categories based on a previous meta-analysis on related topics categorized into subjective well-being (cognitive well-being, affective well-being (positive and negative affect), and psychological well-being^33^. We also included social well-being, drawing on the European Social Survey (ESS) Well-being Module^42^—a systematic way measuring well-being indicators. For the relevance of this meta-analysis, we included the interpersonal feeling category, which conceptualizes social well-being in terms of belonging, social support, social cohesion, social recognition and societal progress. Similarly to^33^ for our third research question on causation, we included experimental or longitudinal studies that manipulated compassion for others or empathic concern and assessed their effects on at least one of five subcategories of well-being. Inclusion criteria encompass interventions e.g. Cognitively Based Compassion Training (CBCT®), Compassion Cultivation Training (CCT®), Mindfulness-based Compassionate Living (MBCL), Compassion-focused Therapy, lovingkindness meditation practices, activities like compassion-based writing, and role-play scenarios that promote compassion for others. Exclusion criteria involved studies that do not target compassion for others or lack measurement of compassion-related or well-being outcomes. Studies with active and non-active controls were included. For trait compassion interventions, control groups could receive alternative interventions such as Cognitive-Behavioral Therapy or non-active controls like treatment as usual. Similarly, state compassion interventions were compared with active or non-active controls, including exercises like expressive writing or standard care protocols, as well as non-active wait-list controls.

### Search strategy

PubMed, PsycINFO, EMBASE, and Web of Science were searched from inception until 15^th^ of July 2024. Grey literature was also included: 1) ProQuest, 2) contacted the mailing list of the German Psychological Society (DGPs), and 3) contacted known researchers in the field. The search string included free terms and index terms for compassion for others and well-being. We also included relevant scales for compassion for others in our search string to increase the number of results. A detailed search strategy and search string can be found in the Supplementary Material. We extracted data on the demographic characteristics, type of well-being, the scale used for well-being, and correlational coefficients between compassion and well-being. Concerning research question 3, we additionally extracted the type of intervention, type of comparison, post-intervention scores on well-being on both groups, and number of participants for each group.

### Study quality

The Quality Appraisal for Diverse Studies (QuADS)^43^ tool was used to assess the quality of the studies. This tool was used as we had mixed studies, correlational for our main research question, and experiments and randomized controlled trials for our additional causal research question. QUADS has 13 categories, which can be rated from 0 to 3. Criteria included aspects from conceptualization to data collection and analysis. Based on prior work^44^, the studies were excluded if they scored lower than the 50% threshold (19.5/39).

### Statistical analysis

All analyses were conducted in R (version 4.4.2) using the metafor package^45^. Pearson r values between compassion for others and well-being were used as effect sizes, with negative affect being reverse coded so that higher values consistently indicated better well-being. Correlation coefficients (r) were then transformed using Fisher’s z-transformation to stabilize variance and normalize distributions. A multilevel meta-analytic model was estimated using the rma.mv() function. Given that multiple effect sizes were reported within several studies, a three-level model was used to account for dependency in the data by modeling random effects at both the study level and the effect size levels^46,47^. The model employed restricted maximum likelihood estimation (REML) and t-distribution-based inference to estimate the average Fisher’s z-transformed effect size, standard errors, confidence intervals, and *p*-values. Heterogeneity was decomposed into three levels: Level 1 (sampling variance), Level 2 (within-study heterogeneity), and Level 3 (between-study heterogeneity). The analysis was repeated after removal of outliers^48^.

We conducted planned moderator analyses using random-effects models to examine theoretically informed sources of variability in effect sizes^49^. Separate multilevel meta-regressions were performed for each of the following study-level moderators: a) well-being type, b) percentage of female participants, c) mean participant age, d) study region (Western vs. Eastern countries). Percentage female and mean age were modeled as continuous moderators. Well-being and region were modeled as categorical dummy-coded moderators. The significance of moderators was evaluated using Wald-type tests (QM or F tests, depending on model structure), and 95% confidence intervals were reported for all parameter estimates. Residual heterogeneity (QE) was assessed after each model to evaluate unexplained variance. All analyses were conducted using restricted maximum likelihood (REML) estimation and t-distributed test statistics.

Potential publication bias and small-study effects were assessed using multiple approaches. Egger’s regression test^50^ was conducted within a multilevel meta-analytic framework that accounted for dependency among effect sizes by modeling random effects at both the study and effect-size levels^45^. In addition, funnel plots were visually inspected to evaluate asymmetry in the distribution of effect sizes relative to study precision.

For the causal research question, we conducted a random-effects meta-analysis to estimate within-group changes in well-being following compassion-based state or trait interventions. Due to the limited availability of between-group data, we included only pre- and post-intervention scores from the intervention groups, even in studies with control conditions. Hedges’ *g* was calculated for dependent samples using an assumed pre–post correlation of *r* = 0.5 and corrected for small sample bias^51^. A REML model was used to pool effects and assess heterogeneity (*I*^*2*^, *τ*^*2*^, Q-statistic).

## Results

**Fig. 1:**
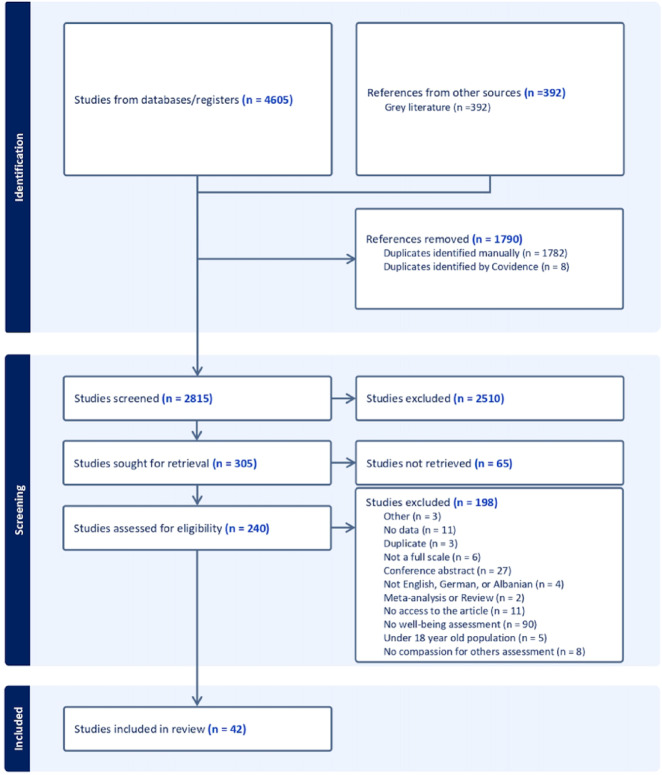
PRISMA flow diagram of study selection.

Search string resulted in a total of 4213 studies, of which 1641 were duplicates, removed manually with Zotero as shown in Fig. 1. We found an additional 8 duplicates from Covidence that were further removed. Two independent reviewers did the title and abstract screening and full-text screening. If conflicts arose, they were solved by a third senior researcher. After title and abstract screening, we removed 2580 studies that did not meet the inclusion criteria. 305 studies were screened for full-text inclusion, of which 263 had to be excluded. We included a total of 42 studies, 37 studies addressing research questions 1 and 2, and 5 studies addressing the causal research question (3). Data extraction was also conducted by two independent reviewers, and if conflicts arose, they were solved by a third senior researcher. Missing data on studies was addressed by contacting the authors.

### Study characteristics

A total of k = 54 effect sizes from 37 studies were extracted for research questions 1 and 2. The total number of participants was 16,013, with a mean age of 32.36 (SD = 9.63) and 66.07% of participants female. The majority of studies (70%) regarding research questions 1 and 2 were conducted in a Western country, while 30% were conducted in an Eastern country. This distribution allowed us to examine Region (Western vs Eastern) as a potential moderator. Regarding the causal research question a total of k = 6 effect sizes from 5 studies were included. Mean age was 33.94 (SD = 7.11) and 75.34% of participants were female. All the studies regarding the causal research question took place in a Western country. The Supplementary Material shows effect sizes and study characteristics in detail for each study (Table S1 and S2).

### Study quality assessment

All studies passed the 50% threshold for quality assessment according to QUADS criteria. The ratings ranged from 25 to 38 out of 39 total scores. The overall mean score was 30.87. The detailed rating of each category for each study can be found in the Supplementary Material (Table S3).

### Overall meta-analysis results

**Fig. 2:**
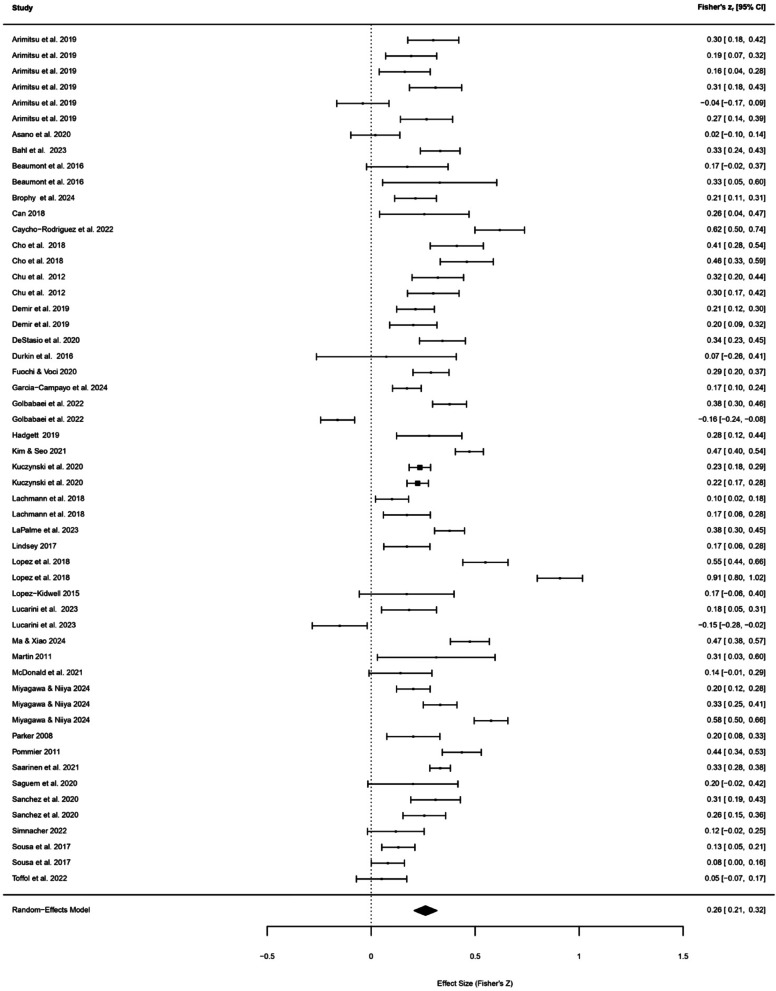
Forest plot of overall effect sizes of compassion for others and well-being.

We conducted a three-level meta-analysis using a random-effects model to account for effect sizes nested within studies. The analysis included k = 54 effect sizes from 37 studies. Fisher’s z-transformed effect sizes were used as the outcome metric, and models were estimated using restricted maximum likelihood (REML). The overall effect of compassion on well-being was positive and statistically significant, z = 0.261, SE = 0.027, 95% CI [0.208, 0.315], *p* < 0.001 as shown in Fig. 2. Back-transformation to Pearson’s *r* indicated an estimated correlation of *r* = 0.255, 95% CI [0.205, 0.305]. After identifying and removing 3 outliers, the results of the overall multilevel meta-analytic effect remained significant, *z* = 0.27 (*r* = 0.26), 95% CI [0.22, 0.31], *p* < 0.001.

### Heterogeneity

There was substantial heterogeneity in the data. The intraclass variance estimates indicated that Level 2 = 66.14% of variability in the model can be accounted for at the within-study level, and Level 3 = 26.33% of variability can be accounted for at the between-study level. The remaining 7.53% of variance was attributable to sampling error. We then compared the three-level model (with random effects for effect sizes nested within studies) to a model with between-study variance fixed to zero. The likelihood ratio test was non-significant, χ^2^(1) = 1.68, *p* = 0.195, suggesting that between-study variance did not significantly improve model fit. However, we retained the full three-level structure to account for the nested data structure and align with best practices in the multilevel meta-analysis^52^.

### Moderation by type of well-being

We examined whether the association between compassion and well-being differed as a function of the type of well-being assessed, namely subjective well-being (cognitive well-being, affective well-being including positive and negative affect), psychological well-being, and social well-being. To test this, a multilevel meta-regression model was estimated using well-being type as a categorical moderator. The model accounted for the hierarchical structure of the data, modeling random effects at the study level and at the effect-size level to account for dependence among multiple effect sizes drawn from the same study. Dummy coding was applied, with psychological well-being serving as the reference category. The full model explained significant variation in effect sizes as a function of well-being type, *F*(4, 49) = 4.89, *p* = 0.002. Compared to psychological well-being, the effect size was significantly weaker for negative affect, β =  − 0.21, *p* = 0.001. There were no significant differences between psychological well-being and cognitive well-being (β =  − 0.10, *p* = 0.105), positive affect (β =  − 0.00, *p* = 0.976), or social well-being (β =  − 0.00, *p* = 0.969). The intercept of the model was significant, *r* = 0.30 (95% CI [0.22, 0.37], *p* < 0.001. To assess the significance of the moderation effect, we conducted an omnibus likelihood ratio test comparing the full model to a reduced model without the moderator variable. The test indicated a statistically significant improvement in model fit when well-being type was included, Δχ^2^(4) = 13.49, *p* = 0.009, supporting the relevance of this moderator. These findings suggest that while compassion is generally associated with higher well-being, the strength of this relationship varies across types of well-being. The association appears to be significantly weaker when well-being is operationalized as reduced negative affect, while the strength of associations does not significantly differ for cognitive, positive, or social well-being relative to psychological well-being.

### Moderation by age, gender, and region

A separate multilevel meta-regression examined whether mean age moderated the association between compassion and well-being (k = 43). Older samples tended to show slightly larger associations, although this effect did not reach significance *F*(1, 41) = 3.11, *p* = 0.078, β = 0.005. Residual heterogeneity remained significant, QE(41) = 560.04, *p* < 0.001.

A multilevel meta-regression examined whether the percentage of female participants in a sample moderated the association between compassion and well-being (*k* = 50). The moderation effect was not statistically significant, *F*(1, 48) = 1.23, *p* = 0.273, β =  − 0.194, indicating no meaningful difference in effect size as a function of gender composition. Residual heterogeneity remained significant, QE(48) = 612.69, *p* < 0.001.

A multilevel meta-regression was also examined if region (Western vs. Eastern) moderated the association (k = 54). The moderator was not statistically significant, *F*(1, 52) = 0.55, *p* = 0.463. Although the association was slightly weaker in Western samples (β =  − 0.045), this difference was small and not statistically reliable, indicating that the strength of the compassion–well-being association did not meaningfully differ by region. Residual heterogeneity remained significant, QE(52) = 639.82, *p* < 0.001. A table with details for all moderators can be found in the Supplementary Material (Table S4).

Although clinical status (clinical vs. non-clinical sample) was a pre-specified moderator of interest, there were not enough studies in the clinical category to permit reliable analysis.

### Publication bias

**Fig. 3:**
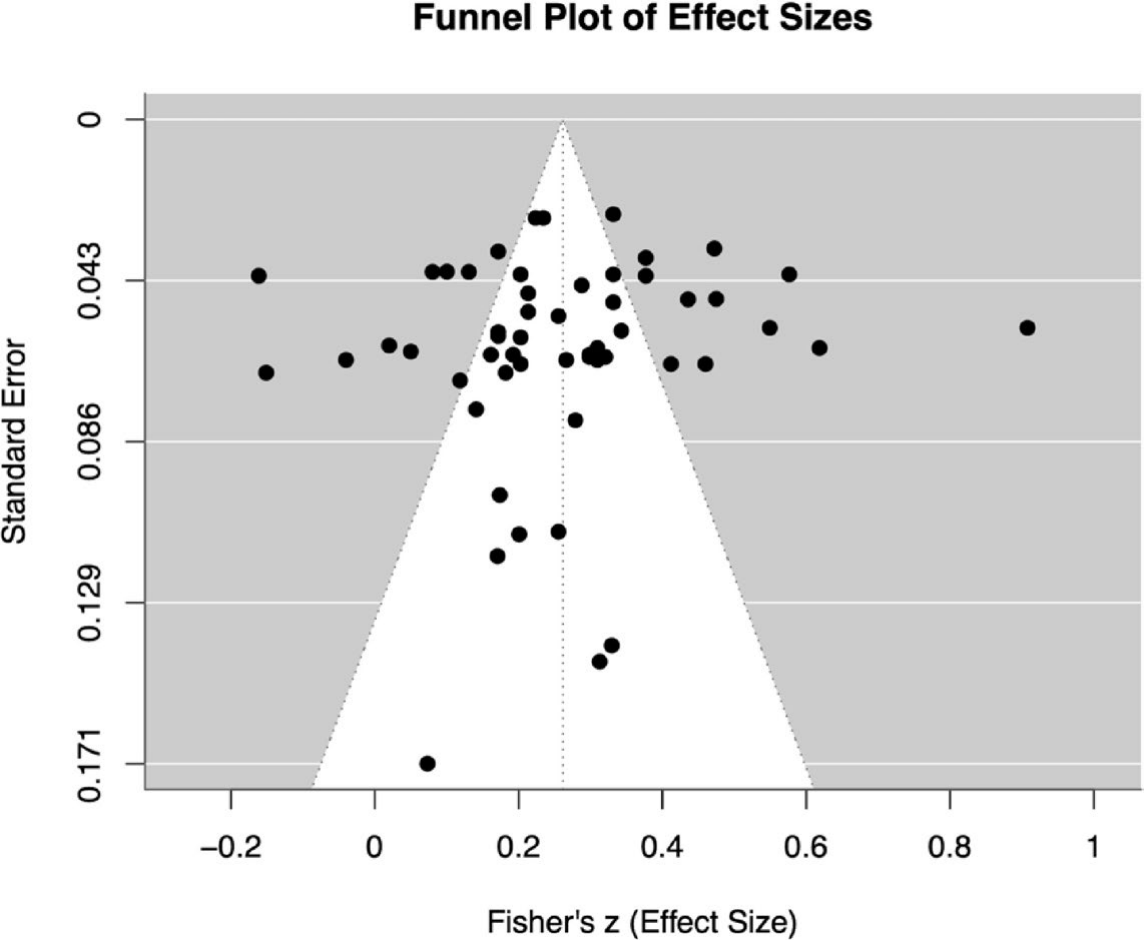
Funnel plot of publication bias.

Visual inspection of the funnel plot revealed a slight degree of asymmetry, with some dispersion toward the right side of the plot as shown in Fig. 3. However, Egger’s test for funnel plot asymmetry was not statistically significant (β = − 0.84, *p* = 0.415), suggesting that evidence for publication bias is limited. Nonetheless, results should be interpreted with caution given residual heterogeneity (QE(52) = 645.01, *p* < 0.001).

### Causal relationship between compassion for others and well-being

Six studies contributed pre-to-post effect sizes from intervention groups. The pooled effect was moderate and statistically significant, g = 0.45, 95% CI [0.29, 0.62], *p* < 0.001. Heterogeneity was low to moderate, I^2^ = 37.1%, τ^2^ = 0.0152, Q(5) = 8.22, *p* = 0.15. While results indicate consistent within-group improvements, they do not allow causal conclusions due to the lack of control group comparisons.

## Discussion

This meta-analysis provides the first comprehensive synthesis of empirical findings on the relationship between compassion for others—a multifaceted construct comprising emotional sensitivity, cognitive understanding, and prosocial motivation^1,2^ and various dimensions of well-being. Our results reveal a moderate, consistent positive association, robust across multiple analytic checks. This extends existing research that has largely emphasized self-compassion^33^, highlighting that compassion directed toward others also contributes meaningfully to well-being, likely via distinct mechanisms.

To contextualize these findings, it is essential to consider the conceptualization of well-being. As discussed in the introduction, subjective and psychological well-being are theoretically and empirically distinct constructs^24^, reflecting the philosophical distinction between hedonic and eudaimonic traditions. The former emphasizes life satisfaction, affect balance, and pleasure^22,25^, whereas the latter centers on meaning, personal growth, and optimal functioning^21,23^. Our moderation analysis indicated that the compassion–well-being link was strongest when well-being was defined in eudaimonic terms, particularly psychological well-being. This supports theoretical propositions suggesting that compassion fosters deeper aspects of flourishing such as meaning-making, self-transcendence, and social connectedness rather than merely alleviating distress^12,26^.

Importantly, the strength of this association varied across well-being types. Stronger effects were found for psychological, social, and cognitive well-being, as well as positive affect, whereas associations with negative affect were weaker. This pattern suggests that compassion for others is more closely linked to the enhancement of positive functioning and interpersonal connectedness than to the reduction of psychological distress. Such differentiation underscores the utility of multidimensional models of well-being in compassion research.

Other potential moderators age, gender, and region, did not significantly alter the observed associations. While minor trends suggested stronger effects for females, older adults, and individuals from Eastern countries, these did not reach statistical significance. Similar demographic patterns have been observed in self-compassion research e.g.,^53–55^, though findings remain context-dependent. These results suggest that the benefits of compassion for others may be broadly generalizable across demographic and cultural boundaries. Nevertheless, cultural theories propose that collectivist values, which are more prevalent in Eastern countries, may place greater value on compassion, potentially enhancing its well-being effects^56^. Likewise, traditional gender roles may increase the social reward of compassion for women, potentially aligning compassionate behavior with well-being-enhancing social expectations^57^.

The robustness of the compassion–well-being link across groups aligns with theoretical perspectives framing compassion as a socially adaptive trait with psychological and physiological benefits. Compassion is known to foster interpersonal connection^12^, reduce stress reactivity^6^, and promote prosocial behavior^13^ mechanisms that plausibly support enhanced well-being, especially within the eudaimonic framework. While cross-sectional data limit causal conclusions, pooled pre-post intervention effects suggest that cultivating compassion for others may lead to moderate improvements in well-being. Meta-analytic evidence on kindness interventions similarly indicates that engaging in prosocial acts produces small to moderate increases in subjective well-being^58^. Neurobiological findings further demonstrate that compassion training can promote neuroplasticity and stress resilience^14,15^. More rigorous longitudinal and experimental research is needed to confirm causal relationships.

This meta-analysis also addresses a critical gap: the relative neglect of compassion for others in favor of self-compassion. Although these constructs are only weakly correlated^38^, they are often conflated. Our findings underscore the need for theoretical and applied models to distinguish clearly between them. Compassion for others independent from self-compassion makes a unique and meaningful contribution to well-being.

Given the robust associations between well-being and broader outcomes such as longevity, health, and social functioning^28,30,31^, promoting compassion for others may represent a promising avenue for psychological and public health interventions. Notably, such interventions may benefit not only recipients but also those expressing compassion reinforcing the reciprocal nature of human flourishing.

Despite the methodological strengths of this meta-analysis including the use of a rigorous multilevel random-effects model, systematic moderator analyses, and a comprehensive quality assessment, several limitations must be acknowledged. First, there was substantial heterogeneity in study design, sample characteristics, and the operationalization of both compassion and well-being. Additionally, the predominant reliance on self-report measures may limit interpretability and generalizability. Second, while tests for publication bias yielded mixed results, small-study effects cannot be ruled out, and some inflation of effect sizes remains possible. Third, although a subset of intervention studies demonstrated moderate within-group improvements in well-being, the absence of control group comparisons limits causal inference. Future work should prioritize randomized controlled trials with adequate follow-up periods.

Fourth, limited data on clinical populations prevented examination of whether the compassion–well-being association differs by mental health status. Given the potential relevance for individuals with elevated psychological distress, this remains an important direction for future research. Additionally, while age was included as a moderator, the cross-sectional nature of the data limits conclusions about developmental change. Longitudinal studies are needed to examine how the compassion well-being link may evolve over the life course.

Finally, the mechanisms underlying the benefits of compassion for others remain poorly understood. Future studies should investigate mediating pathways such as increased social connectedness, enhanced meaning, or improved emotion regulation^59^. Clarifying these mechanisms will strengthen theoretical models and support the development of targeted interventions.

In sum, this meta-analysis provides the first rigorous synthesis of evidence linking compassion for others to well-being, demonstrating a moderate and consistent positive association, especially for eudaimonic outcomes. Although causal conclusions cannot be drawn, the consistent association across studies indicates that compassion for others is reliably aligned with human flourishing. The relationship may be bi-directional, as higher well-being can also encourage compassionate responding^60^. While effects appear generalizable across demographic and cultural contexts, future research should address existing methodological limitations, investigate underlying mechanisms, and explore clinical applications. Compassion for others thus emerges as a promising, yet underutilized, target for well-being-oriented interventions.

## Supplementary Information

Below is the link to the electronic supplementary material.

Supplementary Material 1

Supplementary Material 2

Supplementary Material 3

Supplementary Material 4

## Data Availability

The data used in this meta-analysis is available in the Supplementary Material.
