# Supplementary material for: Compassion for others and well-being: a meta-analysis
Source: Sci Rep. 2025 Oct 20;15:36478. doi: 10.1038/s41598-025-23460-7 (PMC12537975; doi:10.1038/s41598-025-23460-7)
Supplement: Supplementary file 4 — Supplementary Material 4 [file 41598_2025_23460_MOESM4_ESM.pdf]

Supplementary Material
Compassion for Others and Well-being Meta-Analysis

Search Strategy 2
Search String 5
Characteristics of Included Studies 8
QUADS Scores 14
Moderator Analysis 17
References of Included Studies 18

Search strategy

PUBMED

**#1**	(("compassion for other*"[tiab] OR "empathic concern*"[tiab] OR Compassion Motivation Action Scale[tiab] OR Sussex-Oxford Compassion for Others Scale[tiab] OR "Compassionate Love Scale"[tiab] OR "Santa Clara Brief Compassion Scale"[tiab] OR "Interpersonal Reactivity Index"[tiab] OR "Compassion Scale"[tiab] OR Compassionate Engagement and Action Scale[tiab] OR Relational Compassion Scale[tiab] OR The Compassion Scale[tiab] OR The Compassion of Others Lives Scale[tiab] OR Compassion for Other* Scale[tiab] OR "Compassionate Actions"[tiab] OR Compassion towards Other*[tiab] OR "Compassion to other*"[tiab])
**#2**	("Psychological Well-Being"[Mesh] OR "Quality of Life"[Mesh] OR "Happiness"[Mesh] OR "Emotion*"[Mesh] OR "Personal Satisfaction"[Mesh] OR "Mental Healing/psychology"[Mesh] OR "Adaptation, Psychological"[Mesh] OR "well-being"[tiab] OR wellbeing[tiab] OR "cognitive well-being"[tiab] OR "cognitive wellbeing"[tiab] OR "life satisfaction"[tiab] OR "positive evaluation"[tiab] OR "affective well-being"[tiab] OR "affective wellbeing"[tiab] OR "positive emotion*"[tiab] OR "negative emotion*"[tiab] OR "positive affect"[tiab] OR "negative affect"[tiab] OR happiness[tiab] OR "emotional well-being"[tiab] OR "emotional wellbeing"[tiab] OR "psychological well-being"[tiab] OR "psychological wellbeing"[tiab] OR euda?monic wellbeing[tiab] OR euda?monic well-being[tiab] OR eud?monism[tiab] OR euda?monia[tiab] OR "hedonic well-being"[tiab] OR "hedonic wellbeing"[tiab] OR "hedonia"[tiab] OR "hedonic"[tiab] OR "positive mental health"[tiab] OR "social support"[tiab] OR belonging[tiab] OR "social recognition"[tiab] OR "Social Cohesion"[Mesh] OR "Social Support"[Mesh] OR "societal progress"[tiab] OR "social cohesion"[tiab]))
**#3**	**#1 AND #2**

PSYCINFO

**#1**	((TI "Compassion for Other*" OR AB "Compassion for Other*" OR KW "Compassion for Other*") OR (TI "Empathic Concern*" OR AB "Empathic Concern*"OR KW "Empathic Concern*") OR (TI "Compassion Motivation Action Scale" OR AB "Compassion Motivation Action Scale" OR KW "Compassion Motivation Action Scale") OR (TI "Sussex-Oxford Compassion for Others Scale" OR AB "Sussex-Oxford Compassion for Others Scale" OR KW "Sussex-Oxford Compassion for Others Scale") OR (TI "Compassionate Love Scale" OR AB "Compassionate Love Scale" OR KW Compassionate Love Scale") OR (TI "Santa Clara Brief Compassion Scale" OR AB "Santa Clara Brief Compassion Scale" OR ((TI "Compassion for Other*" OR AB "Compassion for Other*" OR KW "Compassion for Other*") OR (TI "Empathic Concern*" OR AB "Empathic Concern*" OR KW "Empathic Concern*") OR (TI "Compassion Motivation Action Scale" OR AB "Compassion Motivation Action Scale" OR KW "Compassion Motivation Action Scale") OR (TI "Sussex-Oxford Compassion for Others Scale" OR AB "Sussex-Oxford Compassion for Others Scale" OR KW "Sussex-Oxford Compassion for Others Scale") OR (TI "Compassionate Love Scale" OR AB "Compassionate Love Scale" OR KW "Compassionate Love Scale") OR (TI "Santa Clara Brief Compassion Scale" OR AB "Santa Clara Brief Compassion Scale" OR KW "Santa Clara Brief Compassion Scale") OR (TI "Interpersonal Reactivity Index" OR AB "Interpersonal Reactivity Index" OR KW "Interpersonal Reactivity Index") OR (TI "Compassion Scale" OR AB "Compassion Scale" OR KW "Compassion Scale") OR (TI "Compassionate Engagement and Action Scale" OR AB "Compassionate Engagement and Action Scale" OR KW "Compassionate Engagement and Action Scale") OR (TI "Relational Compassion Scale" OR AB "Relational Compassion Scale" OR KW "Relational Compassion Scale") OR (TI "The Compassion Scale" OR AB "The Compassion Scale" OR KW "The Compassion Scale") OR (TI "The Compassion of Others Lives Scale" OR AB "The Compassion of Others Lives Scale" OR KW "The Compassion of Others Lives Scale") OR (TI "Compassion for Other* Scale" OR AB "Compassion for Other* Scale" OR KW "Compassion for Other* Scale") OR (TI "Compassionate Action*" OR AB "Compassionate Action*" OR KW "Compassionate Action*") OR (TI "Compassion Towards Other*" OR AB "Compassion Towards

	Other*" OR KW "Compassion Towards Other*") OR (TI "Compassion to Other*" OR AB "Compassion to Other*" OR KW "Compassion to Other*"))
**#2**	((DE "Well Being" OR DE "Emotional States" OR DE "Happiness" OR DE "Life Satisfaction" OR DE "Mental Health" OR DE "Self-Evaluation" OR DE "Self-Perception" OR DE "Contentment" OR DE "Subjective Well Being" OR DE "Hedonism" OR DE "Positive Emotions" OR DE "Negative Emotions" OR DE "Social Support" OR DE "Perceived Social Support" OR DE "Social Connectedness" OR DE "Belonging" OR DE "Social Cohesion") OR (TI (well-being OR wellbeing OR cognitive wellbeing OR cognitive well-being OR life satisfaction OR positive evaluation OR affective wellbeing OR affective well-being OR positive emotion* OR negative emotion* OR positive affect OR negative affect OR happiness OR emotional well-being OR emotional wellbeing OR psychological well-being OR psychological wellbeing OR positive mental health OR hedonic well-being OR hedonic wellbeing OR euda?monic wellbeing OR euda?monic well-being OR eudaimonism OR hedonism OR social support OR belonging OR social recognition OR societal progress OR social cohesion) OR AB (well-being OR wellbeing OR cognitive wellbeing OR cognitive well-being OR life satisfaction OR positive evaluation OR affective wellbeing OR affective well-being OR positive emotion* OR negative emotion* OR positive affect OR negative affect OR happiness OR emotional well-being OR emotional wellbeing OR psychological well-being OR psychological wellbeing OR positive mental health OR hedonic well-being OR hedonic wellbeing OR euda?monic wellbeing OR euda?monic well-being OR eudaimonism OR hedonism OR social support OR belonging OR social recognition OR societal progress OR social cohesion) OR KW (well-being OR wellbeing OR cognitive wellbeing OR cognitive well-being OR life satisfaction OR positive evaluation OR affective wellbeing OR affective well-being OR positive emotion* OR negative emotion* OR positive affect OR negative affect OR happiness OR emotional well-being OR emotional wellbeing OR psychological well-being OR psychological wellbeing OR positive mental health OR hedonic well-being OR hedonic wellbeing OR euda?monic wellbeing OR euda?monic well-being OR eudaimonism OR hedonism OR social support OR belonging OR social recognition OR societal progress OR social cohesion)))
**#3**	**#1 AND #2**

EMBASE

**#1**	('compassion for other*':ab,ti,kw OR 'empathic concern*':ab,ti,kw OR 'compassion motivation action scale':ab,ti,kw OR 'sussex-oxford compassion for others scale':ab,ti,kw OR 'compassionate love scale':ab,ti,kw OR 'santa clara brief compassion scale':ab,ti,kw OR 'interpersonal reactivity index':ab,ti,kw OR 'compassion scale':ab,ti,kw OR 'compassionate engagement and action scale':ab,ti,kw OR 'relational compassion scale':ab,ti,kw OR 'the compassion scale':ab,ti,kw OR 'the compassion of others lives scale':ab,ti,kw OR 'compassion for other* scale':ab,ti,kw OR 'compassionate actions':ab,ti,kw OR 'compassion towards other*':ab,ti,kw OR 'compassion to other*':ab,ti,kw)
**#2**	('psychological well-being'/exp OR 'quality of life'/exp OR 'happiness'/exp OR 'emotion*' OR 'satisfaction'/exp OR 'psychological aspect'/de OR 'psychological adjustment'/exp OR 'well-being' OR 'psychological well-being':ab,ti,kw OR 'quality of life':ab,ti,kw OR 'happiness':ab,ti,kw OR 'emotion*':ab,ti,kw OR 'satisfaction':ab,ti,kw OR 'psychological aspect':ab,ti,kw OR 'psychological adjustment':ab,ti,kw OR 'well-being':ab,ti,kw OR 'wellbeing':ab,ti,kw OR 'cognitive well-being':ab,ti,kw OR 'cognitive wellbeing':ab,ti,kw OR 'life satisfaction':ab,ti,kw OR 'positive evaluation':ab,ti,kw OR 'affective well-being':ab,ti,kw OR 'affective wellbeing':ab,ti,kw OR 'positive emotion*':ab,ti,kw OR 'negative emotion*':ab,ti,kw OR 'positive affect':ab,ti,kw OR 'negative affect':ab,ti,kw OR 'happiness':ab,ti,kw OR 'emotional well-being':ab,ti,kw OR 'emotional wellbeing':ab,ti,kw OR 'eudaimonic wellbeing':ab,ti,kw OR 'eudaemonic wellbeing':ab,ti,kw OR 'eudaemonic wellbeing':ab,ti,kw OR 'eudaimonic wellbeing':ab,ti,kw OR 'eudaimonic well-being':ab,ti,kw OR 'eudaemonic well-being':ab,ti,kw OR 'eudimonism':ab,ti,kw OR

	'eudaimonia':ab,ti,kw OR 'hedonic well-being':ab,ti,kw OR 'hedonic wellbeing':ab,ti,kw OR 'hedonia':ab,ti,kw OR 'hedonic':ab,ti,kw OR 'positive mental health':ab,ti,kw OR 'social support'/exp OR 'belongingness'/exp OR 'social cohesion'/exp OR 'social connectedness'/exp OR 'social support':ab,ti,kw OR 'belonging':ab,ti,kw OR 'social recognition':ab,ti,kw OR 'societal progress':ab,ti,kw OR 'social cohesion':ab,ti,kw)
**#3**	**#1 AND #2**

WEB OF SCIENCE

**#1**	TS=("Compassion for Other*" OR "Empathic Concern*" OR Compassion Motivation Action Scale OR Sussex-Oxford Compassion for Others Scale OR "Compassionate Love Scale" OR "Santa Clara Brief Compassion Scale" OR "Interpersonal Reactivity Index" OR "Compassion Scale" OR Compassionate Engagement and Action Scale OR Relational Compassion Scale OR The Compassion Scale OR The Compassion of Others Lives Scale OR Compassion for other* Scale OR "Compassionate Action*" OR Compassion Towards Other*OR "Compassion to Other*")
**#2**	TS=(well-being OR wellbeing OR cognitive wellbeing OR cognitive well-being OR life satisfaction OR positive evaluation OR affective wellbeing OR affective well-being OR positive emotion* OR negative emotion* OR positive affect OR negative affect OR happiness OR emotional well-being OR emotional wellbeing OR psychological well-being OR psychological wellbeing OR positive mental health OR hedonic well-being OR hedonic wellbeing OR eudemonic wellbeing OR eudaemonic well-being OR eudaimonism OR hedonism OR social support OR belonging OR social recognition OR societal progress OR social cohesion)
**#3**	**#1 AND #2**

PROQUEST

**#1**	TI,AB("compassion for other*" OR ("empathic concern") OR "Compassion Motivation Action Scale" OR "Sussex-Oxford Compassion for Others Scale" OR "Compassionate Love Scale" OR "Santa Clara Brief Compassion Scale" OR "Interpersonal Reactivity Index" OR "Compassion Scale" OR "Compassionate Engagement and Action Scale" OR "Relational Compassion Scale" OR "The Compassion Scale" OR "The Compassion of Others Lives Scale" OR "Compassion for Other* Scale" OR "Compassionate Actions" OR "Compassion towards Other*" OR "Compassion to other*")
**#2**	TI,AB("Psychological Well-Being" OR "Quality of Life" OR "Happiness" OR "Emotion*" OR "Personal Satisfaction" OR "Mental Healing/psychology" OR "Adaptation, Psychological" OR "well-being" OR wellbeing OR "cognitive well-being" OR "cognitive wellbeing" OR "life satisfaction" OR "positive evaluation" OR "affective well-being" OR "affective wellbeing" OR ("positive emotion" OR "positive emotional" OR "positive emotions") OR ("negative emotion" OR "negative emotional" OR "negative emotionality" OR "negative emotions") OR "positive affect" OR "negative affect" OR happiness OR "emotional well-being" OR "emotional wellbeing" OR "psychological well-being" OR "psychological wellbeing" OR eudaemonic wellbeing OR eudaemonic well-being OR eudaemonism OR eudaimonia OR "hedonic well-being" OR "hedonic wellbeing" OR hedonic OR hedonic OR "positive mental health" OR "social support" OR belonging OR "social recognition" OR "Social Cohesion" OR "Social Support" OR "societal progress" OR "social cohesion")
**#3**	**#1 AND #2**

Search String

PUBMED
(("compassion for other*"[tiab] OR "empathic concern*"[tiab] OR Compassion Motivation Action Scale[tiab] OR Sussex-Oxford Compassion for Others Scale[tiab] OR "Compassionate Love Scale"[tiab] OR "Santa Clara Brief Compassion Scale"[tiab] OR "Interpersonal Reactivity Index"[tiab] OR "Compassion Scale"[tiab] OR Compassionate Engagement and Action Scale[tiab] OR Relational Compassion Scale[tiab] OR The Compassion Scale[tiab] OR The Compassion of Others Lives Scale[tiab] OR Compassion for Other* Scale[tiab] OR "Compassionate Actions"[tiab] OR Compassion towards Other*[tiab] OR "Compassion to other*"[tiab]) ("Psychological Well-Being"[Mesh] OR "Quality of Life"[Mesh] OR "Happiness"[Mesh] OR "Emotion*"[Mesh] OR "Personal Satisfaction"[Mesh] OR "Mental Healing/psychology"[Mesh] OR "Adaptation, Psychological"[Mesh] OR "well-being"[tiab] OR wellbeing[tiab] OR "cognitive well-being"[tiab] OR "cognitive wellbeing"[tiab] OR "life satisfaction"[tiab] OR "positive evaluation"[tiab] OR "affective well-being"[tiab] OR "affective wellbeing"[tiab] OR "positive emotion*"[tiab] OR "negative emotion*"[tiab] OR "positive affect"[tiab] OR "negative affect"[tiab] OR happiness[tiab] OR "emotional well-being"[tiab] OR "emotional wellbeing"[tiab] OR "psychological well-being"[tiab] OR "psychological wellbeing"[tiab] OR euda?monic wellbeing[tiab] OR euda?monic well-being[tiab] OR eud?monism[tiab] OR euda?monia[tiab] OR "hedonic well-being"[tiab] OR "hedonic wellbeing"[tiab] OR "hedonia"[tiab] OR "hedonic"[tiab] OR "positive mental health"[tiab] OR "social support"[tiab] OR belonging[tiab] OR "social recognition"[tiab] OR "Social Cohesion"[Mesh] OR "Social Support"[Mesh] OR "societal progress"[tiab] OR "social cohesion"[tiab]))

PSYCINFO
((TI "Compassion for Other*" OR AB "Compassion for Other*" OR KW "Compassion for Other*") OR (TI "Empathic Concern*" OR AB "Empathic Concern*" OR KW "Empathic Concern*") OR (TI "Compassion Motivation Action Scale" OR AB "Compassion Motivation Action Scale" OR KW "Compassion Motivation Action Scale") OR (TI "Sussex-Oxford Compassion for Others Scale" OR AB "Sussex-Oxford Compassion for Others Scale" OR KW "Sussex-Oxford Compassion for Others Scale") OR (TI "Compassionate Love Scale" OR AB "Compassionate Love Scale" OR KW "Compassionate Love Scale") OR (TI "Santa Clara Brief Compassion Scale" OR AB "Santa Clara Brief Compassion Scale" OR KW "Santa Clara Brief Compassion Scale") OR (TI "Interpersonal Reactivity Index" OR AB "Interpersonal Reactivity Index" OR KW "Interpersonal Reactivity Index") OR (TI "Compassion Scale" OR AB "Compassion Scale" OR KW "Compassion Scale") OR (TI "Compassionate Engagement and Action Scale" OR AB "Compassionate Engagement and Action Scale" OR KW "Compassionate Engagement and Action Scale") OR (TI "Relational Compassion Scale" OR AB "Relational Compassion Scale" OR KW "Relational Compassion Scale") OR (TI "The Compassion Scale" OR AB "The Compassion Scale" OR KW "The Compassion Scale") OR (TI "The Compassion of Others Lives Scale" OR AB "The Compassion of Others Lives Scale" OR KW "The Compassion of Others Lives Scale") OR (TI "Compassion for Other* Scale" OR AB "Compassion for Other* Scale" OR KW "Compassion for Other* Scale") OR (TI "Compassionate Action*" OR AB "Compassionate Action*" OR KW "Compassionate Action*") OR (TI "Compassion Towards Other*" OR AB "Compassion Towards Other*" OR KW "Compassion Towards Other*") OR (TI "Compassion to Other*" OR AB "Compassion to Other*" OR KW "Compassion to Other*")) AND ((DE "Well Being" OR DE "Emotional States" OR DE "Happiness" OR DE "Life Satisfaction" OR DE "Mental Health" OR DE "Self-Evaluation" OR DE "Self-Perception" OR DE "Contentment" OR DE "Subjective Well Being" OR DE "Hedonism" OR DE "Positive Emotions" OR DE "Negative Emotions" OR DE "Social Support" OR DE "Perceived Social Support" OR DE "Social Connectedness" OR DE "Belonging" OR DE "Social Cohesion") OR (TI (well-being OR wellbeing OR cognitive wellbeing OR cognitive well-being OR life satisfaction OR positive evaluation OR affective wellbeing OR affective well-being OR positive emotion* OR negative emotion* OR positive affect OR negative affect OR happiness OR emotional well-being OR emotional wellbeing OR psychological well-being OR psychological wellbeing OR positive mental health OR hedonic well-being OR hedonic wellbeing OR euda?monic wellbeing OR euda?monic well-being OR eudaimonism OR hedonism OR social support OR belonging OR social recognition OR societal progress OR social cohesion) OR AB (well-being OR wellbeing OR cognitive wellbeing OR cognitive well-being OR life satisfaction OR positive evaluation OR affective wellbeing OR affective well-being OR positive emotion* OR negative emotion* OR positive affect OR negative affect OR happiness OR emotional well-being OR emotional wellbeing OR psychological well-being OR psychological wellbeing OR positive mental health OR hedonic well-being OR hedonic wellbeing OR euda?monic wellbeing OR euda?monic well-being OR eudaimonism OR hedonism OR social support OR belonging OR social recognition OR societal progress OR social cohesion) OR KW (well-being OR wellbeing OR cognitive

wellbeing OR cognitive well-being OR life satisfaction OR positive evaluation OR affective wellbeing OR affective well-being OR positive emotion* OR negative emotion* OR positive affect OR negative affect OR happiness OR emotional well-being OR emotional wellbeing OR psychological well-being OR psychological wellbeing OR positive mental health OR hedonic well-being OR hedonic wellbeing OR euda?monic wellbeing OR euda?monic well-being OR eudaimonism OR hedonism OR social support OR belonging OR social recognition OR societal progress OR social cohesion)))

EMBASE
('compassion for other*':ab,ti,kw OR 'empathic concern*':ab,ti,kw OR 'compassion motivation action scale':ab,ti,kw OR 'sussex-oxford compassion for others scale':ab,ti,kw OR 'compassionate love scale':ab,ti,kw OR 'santa clara brief compassion scale':ab,ti,kw OR 'interpersonal reactivity index':ab,ti,kw OR 'compassion scale':ab,ti,kw OR 'compassionate engagement and action scale':ab,ti,kw OR 'relational compassion scale':ab,ti,kw OR 'the compassion scale':ab,ti,kw OR 'the compassion of others lives scale':ab,ti,kw OR 'compassion for other* scale':ab,ti,kw OR 'compassionate actions':ab,ti,kw OR 'compassion towards other*':ab,ti,kw OR 'compassion to other*':ab,ti,kw) AND ('psychological well-being'/exp OR 'quality of life'/exp OR 'happiness'/exp OR 'emotion*' OR 'satisfaction'/exp OR 'psychological aspect'/de OR 'psychological adjustment'/exp OR 'well-being' OR 'psychological well-being':ab,ti,kw OR 'quality of life':ab,ti,kw OR 'happiness':ab,ti,kw OR 'emotion*':ab,ti,kw OR 'satisfaction':ab,ti,kw OR 'psychological aspect':ab,ti,kw OR 'psychological adjustment':ab,ti,kw OR 'well-being':ab,ti,kw OR 'wellbeing':ab,ti,kw OR 'cognitive well-being':ab,ti,kw OR 'cognitive wellbeing':ab,ti,kw OR 'life satisfaction':ab,ti,kw OR 'positive evaluation':ab,ti,kw OR 'affective well-being':ab,ti,kw OR 'affective wellbeing':ab,ti,kw OR 'positive emotion*':ab,ti,kw OR 'negative emotion*':ab,ti,kw OR 'positive affect':ab,ti,kw OR 'negative affect':ab,ti,kw OR 'happiness':ab,ti,kw OR 'emotional well-being':ab,ti,kw OR 'emotional wellbeing':ab,ti,kw OR 'eudaimonic wellbeing':ab,ti,kw OR 'eudaemonic wellbeing':ab,ti,kw OR 'eudaemonic wellbeing':ab,ti,kw OR 'eudaimonic wellbeing':ab,ti,kw OR 'eudaimonic well-being':ab,ti,kw OR 'eudaemonic well-being':ab,ti,kw OR 'eudimonism':ab,ti,kw OR 'eudaimonia':ab,ti,kw OR 'hedonic well-being':ab,ti,kw OR 'hedonic wellbeing':ab,ti,kw OR 'hedonia':ab,ti,kw OR 'hedonic':ab,ti,kw OR 'positive mental health':ab,ti,kw OR 'social support'/exp OR 'belongingness'/exp OR 'social cohesion'/exp OR 'social connectedness'/exp OR 'social support':ab,ti,kw OR 'belonging':ab,ti,kw OR 'social recognition':ab,ti,kw OR 'societal progress':ab,ti,kw OR 'social cohesion':ab,ti,kw)

WEB OF SCIENCE
TS=("Compassion for Other*" OR "Empathic Concern*" OR "Compassion Motivation Action Scale" OR "Sussex-Oxford Compassion for Others Scale" OR "Compassionate Love Scale" OR "Santa Clara Brief Compassion Scale" OR "Interpersonal Reactivity Index" OR "Compassion Scale" OR "Compassionate Engagement and Action Scale" OR "Relational Compassion Scale" OR "The Compassion Scale" OR "The Compassion of Others Lives Scale" OR "Compassion for Other* Scale" OR "Compassionate Action*" OR "Compassion Towards Other*" OR "Compassion to Other*") AND TS=("well-being" OR "wellbeing" OR "cognitive wellbeing" OR "cognitive well-being" OR "life satisfaction" OR "positive evaluation" OR "affective wellbeing" OR "affective well-being" OR "positive emotion*" OR "negative emotion*" OR "positive affect" OR "negative affect" OR "happiness" OR "emotional well-being" OR "emotional wellbeing" OR "psychological well-being" OR "psychological wellbeing" OR "positive mental health" OR "hedonic well-being" OR "hedonic wellbeing" OR "euda?monic wellbeing" OR "euda?monic well-being" OR "eudaimonism" OR "hedonism" OR "social support" OR "belonging" OR "social recognition" OR "societal progress" OR "social cohesion")

PROQUEST

TI,AB("compassion for other*" OR ("empathic concern") OR "Compassion Motivation Action Scale" OR "Sussex-Oxford Compassion for Others Scale" OR "Compassionate Love Scale" OR "Santa Clara Brief Compassion Scale" OR "Interpersonal Reactivity Index" OR "Compassion Scale" OR "Compassionate Engagement and Action Scale" OR "Relational Compassion Scale" OR "The Compassion Scale" OR "The Compassion of Others Lives Scale" OR "Compassion for Other* Scale" OR "Compassionate Actions" OR "Compassion towards Other*" OR "Compassion to other*") AND

TI,AB("Psychological Well-Being" OR "Quality of Life" OR "Happiness" OR "Emotion*" OR "Personal Satisfaction" OR "Mental Healing/psychology" OR "Adaptation, Psychological" OR "well-being" OR wellbeing OR "cognitive well-being" OR "cognitive wellbeing" OR "life satisfaction" OR "positive evaluation" OR "affective well-being" OR "affective wellbeing" OR ("positive emotion" OR "positive emotional" OR "positive emotions") OR ("negative emotion" OR "negative emotional" OR "negative emotionality" OR "negative

emotions") OR "positive affect" OR "negative affect" OR happiness OR "emotional well-being" OR "emotional wellbeing" OR "psychological well-being" OR "psychological wellbeing" OR eudaemonic wellbeing OR eudaemonic well-being OR eudaemonism OR eudaimonia OR "

Table S1

Characteristics of Included Studies

Author, Year	Study ID	Effect Size ID	Country	Sample Size	Effect Size	Well-being Instrument	Well-being Type	Percentage Female	*M* Age
Arimitsu et al. (2019)	1a	1	W	258	0,29	PANAS	PA	59%	37,25
Arimitsu et al. (2019)	1a	2	W	258	-0,19	PANAS	NA	59%	37,25
Arimitsu et al. (2019)	1a	3	W	258	0.16	SWLS	CWB	59%	37,25
Arimitsu et al. (2019)	1b	4	E	247	0,30	PANAS	PA	55%	36,50
Arimitsu et al. (2019)	1b	5	E	247	0,04	PANAS	NA	55%	36,50
Arimitsu et al. (2019)	1b	6	E	247	0,26	SWLS	CWB	55%	36,50
Asano et al. (2020)	2	7	E	279	0,02	SWLS	CWB	68,45%	19,35
Bahl et al. (2023)	3	8	W	425	0,32	SWLS	CWB	48,11%	
Beaumont et al. (2016)*	4	9	W	103	0,17	SWEMWBS	PWB	100%	
Beaumont et al. (2016)**	5	10	W	54	0,32	SWEMWBS	PWB		
Brophy et al. (2024)	6	11	W	384	0,21	WEMWBS	PWB	86,79%	34,97
Can (2018)	7	12	W	86	0,25	FS	PWB	90,70%	32,89
Caycho-Rodriguez et al. (2022)	8	13	E	273	0,55	WHO-5	PWB	50,90%	21,23
Cho et al. (2018)	9	14	E	239	0,39	SWLS	CWB		
Cho et al. (2018)	9	15	E	239	0,43	SCS-R	SWB		
Chu et al. (2012)	10	16	W	253	0,31	PANAS	PA	63%	
Chu et al. (2012)	10	17	W	253	-0,29	PANAS	NA	63%	

Author, Year	Study ID	Effect Size ID	Country	Sample Size	Effect Size	Well-being Instrument	Well-being Type	Percentage Female	*M* Age
Demir et al. (2019)	11a	18	W	470	0,21	SWLS	CWB	80,43%	18,90
Demir et al. (2019)	11b	19	W	300	0,20	PANAS	PA	67%	18,84
DeStasio et al. (2020)	12	20	W	319	0,33	SHS	PWB	92,70%	42,26
Durkin et al. (2016)	13	21	W	37	0,07	SWEMWBS	PWB	91,89%	36,00
Fuochi & Voci (2020) Study 3	14	22	W	516	0,28	SCS	SWB	53%	29,03
Garcia-Campayo et al. (2024)	15	23	W	811	0,17	SWEMWBS	PWB	80%	43,49
Golbabaei et al. (2022) Study 1	16	24	E	580	0,36	PANAS	NA	57,93%	24,52
Golbabaei et al. (2022) Study 1	16	25	E	580	0,16	PANAS	PA	57,93%	24,52
Hadgett (2019)	17	26	W	159	0,27	WEMWBS	PWB	79,20%	
Kim & Seo (2021)	18	27	E	850	0,44	WEMWBS	PWB	50%	43,98
Kuczynski et al. (2020)	19	28	W	1456	0,23	SCS	SWB	62%	19,69
Kuczynski et al. (2020)	19	29	W	1456	0,22	WHOQOL	PWB	62%	19,69
Lachmann et al. (2018)	20a	30	E	612	0,10	SOEP	CWB	26,47%	21,55
Lachmann et al. (2018)	20b	31	W	304	0,17	SOEP	CWB	68,09%	24,05
LaPalme et al. (2023) Study 4	21	32	W	740	0,36	SCS	SWB	48,80%	39,36
Lindsey (2017)	22	33		315	0,17	TPAS	PA	83,50%	39,00
Lopez et al. (2018)	23	34	W	328	-0,50	PANAS-SF	NA	55,20%	57,00

Author, Year	Study ID	Effect Size ID	Country	Sample Size	Effect Size	Well-being Instrument	Well-being Type	Percentage Female	*M* Age
Lopez et al. (2018)	23	35	W	328	0,72	PANAS-SF	PA	55,20%	57,00
Lopez-Kidwell (2015)	24	36	W	77	0,17	PA	PA		34.85
Lucarini et al. (2023)	25	37	W	223	0,18	PANAS-SF	PA	61,43%	31,83
Lucarini et al. (2023)	25	38	W	223	0,15	PANAS-SF	NA	61,43%	31,83
Ma & Xiao (2024)	26	39	E	441	0,44	MSPSS	SWB	50,57%	22,44
Martin (2011)	27	40	W	51	0,30	WEMWBS	PWB	100%	36,80
McDonald et al. (2021)	28	41	W	171	0,14	SWLS	CWB	27%	
Miyagawa & Niiya (2024) Study 2	29	42	E	590	0,52	PWB	PWB	53,73%	40,93
Miyagawa & Niiya (2024) Study 2	29	43	E	590	-0,20	PANAS	NA	53,73%	40,93
Miyagawa & Niiya (2024) Study 2	29	44	E	590	0,32	SWLS	CWB	53,73%	40,93
Parker (2008)	30	45	W	239	0,20	SSQ-6	SWB	63,60%	
Pommier (2011)	31	46	W	439	0,41	SCS	SWB	65,14%	20,06
Saarinen et al. (2021)	32	47	W	1573	-0,32	EAS	NA	56,26%	31,66
Saguem et al. (2020)	33	48	E	85	0,20	SSQ-6	SWB	89,41%	29,40
Sanchez et al. (2020)	34a	49	W	273	0,30	PANAS	PA	69,60%	19,13
Sanchez et al. (2020)	34b	50	W	368	0,25	SHS	PA	67,93%	18,90
Simnacher (2022)	35	51	W	210	0,12	SOB-I	SWB	85%	
Sousa et al. (2017)	36	52	W	610	0,13	PANAS	PA	57,70%	39,22
Sousa et al. (2017)	36	53	W	610	-0,08	PANAS	NA	57,70%	39,22

Author, Year	Study ID	Effect Size ID	Country	Sample Size	Effect Size	Well-being Instrument	Well-being Type	Percentage Female	*M* Age
Toffol et al. (2022)	37	54	W	265	0,05	MSPSS	SWB	78,90%	40,00

Note. * Beaumont et al. (2016) Compassion for others, self-compassion, quality of life and mental well-being measures and their association with compassion fatigue and burnout in student midwives: A quantitative survey. Midwifery. 239-244. doi: 10.1016/j.midw.2015.11.002

** Beaumont et al. (2016). Measuring relationships between self-compassion, compassion fatigue, burnout and well-being in student counsellors and student cognitive behavioural psychotherapists: A quantitative survey. Counselling & Psychotherapy Research, 16(1), 15–23. https://doi.org/10.1002/capr.12054

Column names: E = Eastern; W = Western. Well-being types included: CWB refers to cognitive well-being; PWB to psychological well-being; SWB to social well-being; PA to positive affect, and NA to negative affect. Commonly used scales were the SWLS (Satisfaction With Life Scale), PANAS (Positive and Negative Affect Schedule) and its short form PANAS-SF, SWEMWBS (Short Warwick-Edinburgh Mental Well-being Scale), and WEMWBS (Warwick-Edinburgh Mental Well-being Scale). Additional instruments included the FS (Flourishing Scale), WHO-5 (World Health Organization Well-being Index), WHOQOL (World Health Organization Quality of Life Measure), SHS (Subjective Happiness Scale), SOEP (Socio-Economic Panel Well-being Measure), TPAS (Trait Positive Affect Scale), MSPSS (Multidimensional Scale of Perceived Social Support), SSQ-6 (Social Support Questionnaire–Short Form), SCS-R (Self-Compassion Scale–Revised, used here as a proxy for well-being), EAS (Emotional Approach Scale), and SOB-I (Sense of Belonging Instrument).

Table S2

Characteristics of Included Studies (Causal Research Question)

Study	Study-ID	Effect Size ID	Trait or State Intervention	Country	Intervention
Tendhar et al. (2024)	1	1	State	W	Eight Steps to Great Compassion (ESGC)
Gilbert et al. (2023)	2	2	State	W	Video and Guided Meditation
Gilbert et al. (2023)	2	3	State	W	Video and Guided Meditation
Irons & Heriot-Maitland (2021)	3	4	Trait	W	Compassionate Mind Training (8 week)
Gold (2021)	4	5	Trait	W	Brief Compassionate Mind Training
Nebot-Gresa et al. (2021)	5	6	Trait	W	Attachment-Based Compassion Therapy (ABCT) brief version

Table S2b

Study	*N* Intervention	Control	*N* Control	*N* Total	*M* Pre	*SD*-Pre	*M* Post	*SD*-Post
Tendhar et al. (2024)	92	Pre-Post assessment	92	92	147,32	35	164,72	35
Gilbert et al. (2023)	43	Pre-Post assessment	43	43	42,88	7,02	45	7,29
Gilbert et al. (2023)	43	Pre-Post assessment	43	43	50,3	7,42	54,14	7,3
Irons & Heriot-Maitland (2021)	55	Pre-Post assessment	55	55	43,1	8,11	48,69	8,16
Gold (2021)	26	Psychoeducation	29	55	69,7	12,2	70,5	14,1
Nebot-Gresa et al. (2021)	17	No treatment	43	60	8,3	1	8,9	0,6

Table S2c

Study	Well-being Type	Well-being Instrument	% Female	*M* Age
Tendhar et al. (2024)	PWB	PERMA	86%	20,39
Gilbert et al. (2023)	SWB	SSPS	86%	20,39
Gilbert et al. (2023)	PWB	WEWBS	81%	49,35
Irons & Heriot-Maitland (2021)	PWB	WEWBS	67%	42
Gold (2021)	PA	TPA	66.7%	19,2
Nebot-Gresa et al. (2021)	CWB	PHI	76%	38,8

Note. *Column names:* E = Eastern; W = Western. Well-being types included: CWB refers to cognitive well-being; PWB to psychological well-being; SWB to social well-being; and PA to positive affect. Well-being instruments used across studies were the PERMA (Positive Emotion, Engagement, Relationships, Meaning, and Accomplishment Scale), SSPS (Social Support and Positive States Scale), WEWBS (Warwick-Edinburgh Well-being Scale), TPA (Trait Positive Affect Scale), and PHI (Pemberton Happiness Index).

Table S3

QUADS Scores of Included Studies

	Main Studies													
	Criteria Number													
**Author, Year**	**1**	**2**	**3**	**4**	**5**	**6**	**7**	**8**	**9**	**10**	**11**	**12**	**13**	**Total Score**
Arimitsu et al. (2019)	3	3	1	2	1	1	3	2	2	3	3	0	3	27
Asano et al. (2020)	3	3	2	3	2	3	3	2	2	3	3	2	2	33
Bahl et al. (2023)	3	3	1	2	2	2	3	2	3	3	3	0	3	30
Beaumont et al. (2016)*	3	3	3	2	2	3	3	2	1	3	3	0	3	31
Beaumont et al. (2016)**	3	3	3	2	2	2	3	1	1	1	3	0	3	27
Brophy et al. (2024)	3	3	2	3	2	1	2	3	2	3	3	0	3	30
Can (2018)	3	3	2	2	2	3	3	2	3	3	3	0	3	32
Caycho-Rodriguez et al. (2022)	3	3	2	3	3	3	3	3	3	3	3	2	3	37
Cho et al. (2018)	2	3	2	2	3	1	3	2	2	1	3	0	3	27
Chu et al. (2012)	3	3	3	2	1	2	2	3	2	3	3	0	3	30
Demir (2019)	3	3	2	2	2	2	3	3	3	2	3	0	3	31
DeStasio et al. (2020)	3	3	3	2	2	3	3	2	2	3	3	0	3	32
Durkin et al. (2016)	2	3	3	2	2	3	3	1	1	2	3	0	3	28
Fuochi & Voci (2020)	3	3	1	3	2	3	3	2	2	3	3	0	1	29
Garcia-Campayo et al. (2024)	3	3	1	3	2	2	3	3	3	3	3	0	2	31
Golbabaei et al. (2022)	3	3	3	3	2	1	3	2	1	2	3	0	2	28
Hadgett (2019)	3	3	3	2	2	3	3	2	3	3	3	0	3	33
Kim & Seo (2021)	3	3	2	3	3	3	3	2	2	3	3	0	3	33
Kuczynski et al. (2020)	3	3	2	3	2	2	3	2	3	2	3	0	3	31
Lachmann et al. (2018)	3	3	2	2	1	3	3	3	2	3	3	0	3	31
LaPalme et al. (2023)	3	3	3	3	2	2	3	1	1	3	3	0	2	29
Lindsey (2017)	3	3	3	2	3	3	3	3	3	3	3	0	3	35
Lopez et al. (2018)	3	3	1	2	2	2	3	3	3	2	2	0	3	29
Lopez-Kidwell (2015)	3	3	3	2	2	2	3	2	3	3	3	0	3	32
Lucarini et al. (2023)	3	3	1	2	2	1	3	1	2	3	3	0	3	27

Ma & Xiao (2024)	3	3	3	3	2	2	3	2	2	3	3	0	2	31
Martin (2011)	3	3	3	2	2	3	3	2	3	3	3	0	3	33
McDonald et al. (2021)	3	3	3	2	2	3	3	2	3	3	3	0	3	33
Miyagawa & Niiya (2024)	3	3	2	3	2	1	3	2	3	3	3	0	3	31
Parker (2008)	3	3	2	2	2	3	3	2	3	3	3	0	3	32
Pommier (2011)	3	3	2	2	2	2	3	1	2	2	3	0	3	28
Saarinen et al. (2021)	3	3	3	3	2	3	3	2	3	3	3	0	3	34
Simancher (2022)	3	3	3	2	2	3	2	2	3	3	3	0	3	32
Saguem et al. (2020)	2	3	3	3	2	3	3	2	3	2	3	0	3	31
Sanchez et al. (2020)	3	3	2	1	2	2	3	2	1	1	2	0	3	25
Sousa et al. (2017)	3	3	3	3	3	2	3	3	2	2	3	0	3	33
Toffol et al. (2022)	3	3	3	3	2	2	3	2	3	3	3	0	2	32

Note. * Beaumont et al. (2016) Compassion for others, self-compassion, quality of life and mental well-being measures and their association with compassion fatigue and burnout in student midwives: A quantitative survey. Midwifery. 239-244. doi: 10.1016/j.midw.2015.11.002

** Beaumont et al. (2016). Measuring relationships between self-compassion, compassion fatigue, burnout and well-being in student counsellors and student cognitive behavioural psychotherapists: A quantitative survey. Counselling & Psychotherapy Research, 16(1), 15–23. https://doi.org/10.1002/capr.12054

Column names:1 = Theoretical or conceptual underpinning to the research; 2 = Statement of research aim/s; 3 = Clear description of research setting and target population; 4 = The study design is appropriate to address the stated research aim/s; 5 = Appropriate sampling to address the research aim/s; 6 = Rationale for choice of data collection tool/s; 7 = The format and content of data collection tool is appropriate to address the stated research aim/s; 8 = Description of data collection procedure; 9 = Recruitment data provided; 10 = Justification for analytic method selected; 11 = The method of analysis was appropriate to answer the research aim/s; 12 = Evidence that the research stakeholders have been considered in research design or conduct; 13 = Strengths and limitations critically discussed.

Table 3b

QUADS Scores of Included Studies (Causal Research Question)

Causal Studies														
	Criteria Number													
**Author, Year**	**1**	**2**	**3**	**4**	**5**	**6**	**7**	**8**	**9**	**10**	**11**	**12**	**13**	**Total Score**
Gilbert et al. (2023)	3	3	2	2	2	2	3	2	2	2	3	0	3	29
Gold (2021)	3	3	3	2	2	3	3	3	3	3	3	0	3	34
Irons & Heriot-Maitland (2021)	3	3	2	2	1	2	3	2	1	2	3	0	3	27
Nebot-Gresa et al. (2021)	3	3	2	1	2	2	3	2	2	2	3	0	3	28
Tendhar et al. (2024)	3	3	3	2	2	3	3	2	3	2	3	0	3	32

Note. *Column names*:1 = Theoretical or conceptual underpinning to the research; 2 = Statement of research aim/s; 3 = Clear description of research setting and target population; 4 = The study design is appropriate to address the stated research aim/s; 5 = Appropriate sampling to address the research aim/s; 6 = Rationale for choice of data collection tool/s; 7 = The format and content of data collection tool is appropriate to address the stated research aim/s; 8 = Description of data collection procedure; 9 = Recruitment data provided; 10 = Justification for analytic method selected; 11 = The method of analysis was appropriate to answer the research aim/s; 12 = Evidence that the research stakeholders have been considered in research design or conduct; 13 = Strengths and limitations critically discussed.

Table S4

Moderator Analysis

Moderator	*k*	Intercept / Mean Z (95% CI)	*β* (95% CI)	*r*	*F*	*p*	Level 2	Level 3
Wellbeing Type					F(4,49) = 4.89	0.002 **	0.0098	0.0177
Psychological Well-being	13	0.311 (0.228, 0.392)	reference	0.301				
Cognitive Well-being	12	0.208 (0.085,0.332)	-102 (-0.225, 0.022)	0.206		0.105		
Negative Affect	10	0.093 (-0.034, 0.222)	-0.217 (-0.345, -0.089)	0.094		0.001 ***		
Positive Affect	9	0.308 (0.184, 0.433)	-0.002 (-0.126, 0.122)	0.299		0.976		
Social Well-being	10	0.309 (0.242, 0.376)	-0.001 (-0.069, 0.066)	0.3		0.969		
Age (continuous)	43	0.074 (-0.145, 0.294)	0.006 (-0.001, 0.012)		F(1,41) = 3.11	0.085	0.029	0.005
Gender (continuous)	50	0.381 (0.147, 0.615)	-0.194 (-0.547, 0.158)		F(1,48) = 1.23	0.27	0.023	0.008
Region					F(1,52) = 0.54	0.463	0.022	0.009
Eastern	36	0.294 (0.190, 0.397)	reference	0.286				
Western	16	0.248 (0.123, 0.371)	-0.045 (-0.168, 0.077)	0.243				

Note. k = number of independent samples; N = number of participants; β = regression coefficient relative to the reference category; *r* = back-transformed correlation coefficient; Level 2 = within-study; Level 3=between-study.

References of included studies (Table S1 and Table S2)

Arimitsu, K., Hitokoto, H., Kind, S. & Hofmann, S. G. (2018). Differences in Compassion, Well-being, and Social Anxiety Between Japan and the USA. *Mindfulness*, *10*(5), 854–862. https://doi.org/10.1007/s12671-018-1045-6

Asano, K., Kotera, Y., Tsuchiya, M., Ishimura, I., Lin, S., Matsumoto, Y., Matos, M., Basran, J. & Gilbert, P. (2020). The development of the Japanese version of the compassionate engagement and action scales. *PLoS ONE*, *15*(4), e0230875. https://doi.org/10.1371/journal.pone.0230875

Bahl, S., Milne, G. R. & Swani, K. (2023). An expanded mindful mindset: The role of different skills in stress reduction and life satisfaction. *Journal Of Consumer Affairs*, *57*(2), 821–847. https://doi.org/10.1111/joca.12509

Beaumont, E., Durkin, M., Martin, C. J. H. & Carson, J. (2016a). Compassion for others, self-compassion, quality of life and mental well-being measures and their association with compassion fatigue and burnout in student midwives: A quantitative survey. *Midwifery*, *34*, 239–244. https://doi.org/10.1016/j.midw.2015.11.002

Beaumont, E., Durkin, M., Martin, C. J. H. & Carson, J. (2016b). Measuring relationships between self-compassion, compassion fatigue, burnout and well-being in student counsellors and student cognitive behavioural psychotherapists: a quantitative survey. *Counselling And Psychotherapy Research*, *16*(1), 15–23. https://doi.org/10.1002/capr.12054

Brophy, K., Emery, M., MacDonald, C., Côté, C. I. & Körner, A. (2024). Validation of the compassionate engagement and action scales, compassion scale, and Sussex-Oxford compassion scales in a French-Canadian sample. *PLoS ONE*, *19*(6), e0305776. https://doi.org/10.1371/journal.pone.0305776

Can, N. (2018). *Caring for beginning counselors: the relationship between empathy, supervisory working alliance, resilience, wellness, and compassion fatigue counselors-in-training*. (Doctoral dissertation, Texas A&M University-Corpus Christi). ProQuest Dissertations Publishing. https://www.proquest.com/docview/2037210595

Caycho-Rodríguez, T., Vilca, L. W., Plante, T. G., Carbajal-León, C., Cabrera-Orosco, I., Cadena, C. H. G. & Reyes-Bossio, M. (2020). Spanish version of the Santa Clara Brief Compassion Scale: evidence of validity and factorial invariance in Peru. *Current Psychology*, *41*(7), 4431–4446. https://doi.org/10.1007/s12144-020-00949-0

Cho, H., Noh, S., Park, S., Ryu, S., Misan, V. & Lee, J. (2018). The development and validation of the Lovingkindness-Compassion Scale. *Personality And Individual Differences*, *124*, 141–144. https://doi.org/10.1016/j.paid.2017.12.019

Chu, K. H., Baker, M. A. & Murrmann, S. K. (2011). When we are onstage, we smile: The effects of emotional labor on employee work outcomes. *International Journal Of Hospitality Management*, *31*(3), 915. https://doi.org/10.1016/j.ijhm.2011.10.009

De Stasio, S., Benevene, P., Pepe, A., Buonomo, I., Ragni, B. & Berenguer, C. (2020). The Interplay of Compassion, Subjective Happiness and Proactive Strategies on Kindergarten Teachers' Work Engagement and Perceived Working Environment Fit. *International Journal Of Environmental Research And Public Health*, *17*(13), 4869. https://doi.org/10.3390/ijerph17134869

Demir, M., Haynes, A., Sanchez, M. & Parada, J. C. (2019). Personal Sense of Uniqueness Mediates the Relationship Between Compassion for Others and Subjective Well-Being. *Journal Of Happiness Studies*, *20*(6), 1751–1773. https://doi.org/10.1007/s10902-018-0020-1

Durkin, M., Beaumont, E., Martin, C. J. H. & Carson, J. (2016). A pilot study exploring the relationship between self-compassion, self-judgement, self-kindness, compassion, professional quality of life and wellbeing among UK community nurses. *Nurse Education Today*, *46*, 109–114. https://doi.org/10.1016/j.nedt.2016.08.030

Fuochi, G. & Voci, A. (2020). A deeper look at the relationship between dispositional mindfulness and empathy: Meditation experience as a moderator and dereification processes as mediators. *Personality And Individual Differences*, *165*, 110122. https://doi.org/10.1016/j.paid.2020.110122

García-Campayo, J., Barceló-Soler, A., Martínez-Rubio, D., Navarrete, J., Pérez-Aranda, A., Feliu-Soler, A., Luciano, J. V., Baer, R., Kuyken, W. & Montero-Marin, J. (2023). Exploring the Relationship Between Self-Compassion and Compassion for Others: The Role of Psychological Distress and Wellbeing. *Assessment*, *31*(5), 1038–1051. https://doi.org/10.1177/10731911231203966

Gilbert, P., Basran, J. K., Plowright, P. & Gilbert, H. (2023). Energizing compassion: using music and community focus to stimulate compassion drive and sense of connectedness. *Frontiers in Psychology*, *14*. https://doi.org/10.3389/fpsyg.2023.1150592

Golbabaei, S., Barati, M., Haromi, M. E., Ghazazani, N. & Borhani, K. (2022). Development and construct validation of a short form of the interpersonal reactivity index in Iranian community. *Current Psychology*, *42*(16), 14038–14050. https://doi.org/10.1007/s12144-022-02716-9

Gold, G. S. (2021). *Effects of Brief Compassionate Mind Training on Compassion and Psychological Flexibility*. (Doctoral dissertation, Hofstra University). ProQuest Dissertations Publishing. https://www.proquest.com/openview/f6c1f154ed8166c3014aa2738b9fa581/1?cbl=18750&diss=y&pq-origsite=gscholar

Hadgett, L. F. (2019). *The relationship between compassion, burnout and well-being in teachers and other professionals*. (Doctoral dissertation, University of Hull). University of Hull Research Repository. https://hull-repository.worktribe.com/output/4221877

Irons, C. & Heriot-Maitland, C. (2021). Compassionate Mind Training: An 8-week group for the general public. *Psychology And Psychotherapy Theory Research And Practice*, *94*(3), 443–463. https://doi.org/10.1111/papt.12320

Kim, J. & Seo, J. (2021). Assessing Compassion in Korean Population: Psychometric Properties of the Korean Version of Sussex-Oxford Compassion Scales. *Frontiers in Psychology*, *12*. https://doi.org/10.3389/fpsyg.2021.744481

Kuczynski, A. M., Kanter, J. W. & Robinaugh, D. J. (2020). Differential associations between interpersonal variables and quality-of-life in a sample of college students. *Quality Of Life Research*, *29*(1), 127–139. https://doi.org/10.1007/s11136-019-02298-3

Lachmann, B., Sindermann, C., Sariyska, R. Y., Luo, R., Melchers, M. C., Becker, B., Cooper, A. J. & Montag, C. (2018). The Role of Empathy and Life Satisfaction in Internet and Smartphone Use Disorder. *Frontiers in Psychology*, *9*. https://doi.org/10.3389/fpsyg.2018.00398

LaPalme, M. L., Barsade, S. G., Brackett, M. A. & Floman, J. L. (2023). The Meso-Expression Test (MET): A Novel Assessment of Emotion Perception. *Journal Of Intelligence*, *11*(7), 145. https://doi.org/10.3390/jintelligence11070145

Lindsey, S. (2017). *Examining the Psychometric Properties of the Compassionate Engagement and Action Scales in the General Population*. (Doctoral dissertation, University of Essex). University of Essex Research Repository. http://repository.essex.ac.uk/20473/

López, A., Sanderman, R., Ranchor, A. V. & Schroevers, M. J. (2018). Compassion for Others and Self-Compassion: Levels, Correlates, and Relationship with Psychological Well-being. *Mindfulness*, *9*(1), 325–331. https://doi.org/10.1007/s12671-017-0777-z

Lopez-Kidwell, V. (2015). *The Heart of social networks: the ripple effect of emotional abilities in relational well-being*. (Doctoral dissertation, University of Kentucky). Uknowledge. https://uknowledge.uky.edu/cgi/viewcontent.cgi?article=1002&context=management_etds

Lucarini, A., Fuochi, G. & Voci, A. (2022). A deep dive into compassion. *European Journal Of Psychological Assessment*, *39*(5), 371–384. https://doi.org/10.1027/1015-5759/a000717

Ma, J. & Xiao, Q. (2024). Relationship Between Self-Compassion and Compassion for Others: The Mediated Effect of Perceived Social Support and Psychological Resilience. *Psychological Reports*. https://doi.org/10.1177/00332941241226906

Martin, M. W. (2011). *The development of self-criticism and the effects of self-compassion and mindfulness on the well-being of mothers*. (Doctoral dissertation, University of Warwick). University of Warwick Research Repository. https://wrap.warwick.ac.uk/id/eprint/49352/

McDonald, M. A., Meckes, S. J. & Lancaster, C. L. (2021). Compassion for Oneself and Others Protects the Mental Health of First Responders. *Mindfulness*, *12*(3), 659–671. https://doi.org/10.1007/s12671-020-01527-y

Miyagawa, Y. & Niiya, Y. (2024). Psychometric Validation of the Compassion Scale in Japan (CS-J). *Mindfulness*, *15*(7), 1665–1681. https://doi.org/10.1007/s12671-024-02389-4

Nebot-Gresa, L., Llorens, S., Salanova, M., Coo, C. & Garcia-Campayo, J. (2021). Positive effects and validation of a Brief Intervention Program of Attachment-Based Compassion Therapy. *Terapia PsicolóGica*, *39*(3), 427–444. https://doi.org/10.4067/s0718-48082021000300427

Parker, B. (2008). *The relation between hostility and social support: Investigating potential mediation or moderation by trait forgiveness, attributional style, and trait empathy.* (Doctoral dissertation, West Virginia University). West Virginia University Research Repository. https://researchrepository.wvu.edu/etd/9557

Pommier, E. A. (2011). *The compassion scale*. (Doctoral dissertation, The University of Texas at Austin). Texas ScholarWorks. https://repositories.lib.utexas.edu/handle/2152/ETD-UT-2010-12-2213

Saarinen, A. I. L., Keltikangas-Järvinen, L., Pulkki-Råback, L., Cloninger, C. R., Elovainio, M., Lehtimäki, T., Raitakari, O. & Hintsanen, M. (2023). The relationship of dispositional compassion with well-being: a study with a 15-year prospective follow-up. *The Journal Of Positive Psychology*, *15*(6), 806–820. https://doi.org/10.1080/17439760.2019.1663251

Saguem, B. N., Bouzaâbia, Z., Braham, A. & Nasr, S. B. (2020). Empathic abilities and their mediators in Tunisian psychiatry trainees. *The Journal Of Mental Health Training Education And Practice*, *15*(6), 317–330. https://doi.org/10.1108/jmhtep-05-2020-0033

Sanchez, M., Haynes, A., Parada, J. C. & Demir, M. (2020). Friendship Maintenance Mediates the Relationship Between Compassion for Others and Happiness. *Current Psychology*, *39*(2), 581–592. https://doi.org/10.1007/s12144-017-9779-1

Simnacher, C. E. (2022). *Daily Spiritual Experiences, Empathy, and Sense of Belonging as Predictors of Burnout Among Licensed Counselors*. (Doctoral dissertation, New Orleans Baptist Theological Seminary). ProQuest Dissertations Publishing. https://www.proquest.com/openview/00fb27e3f547476f99b6977153838806/1?cbl=18750&diss=y&pq-origsite=gscholar

Sousa, R., Castilho, P., Vieira, C., Vagos, P. & Rijo, D. (2017). Dimensionality and gender-based measurement invariance of the Compassion Scale in a community sample. *Personality And Individual Differences*, *117*, 182–187. https://doi.org/10.1016/j.paid.2017.06.003

Tendhar, T., Marcotte, M. A., De Mesquita, P. B. & Saikia, M. J. (2024). Online Video-Mediated Compassion Training Program for Mental Health and Well-Being of University Students. *Healthcare*, *12*(10), 1033. https://doi.org/10.3390/healthcare12101033

Toffol, E., Aliverti, E., Idotta, C., Capizzi, G. & Scocco, P. (2022). Are empathy profiles and perceived social support associated with depressive and grief-related symptoms in suicide survivors? *Journal Of Clinical Psychology*, *78*(11), 2245–2259. https://doi.org/10.1002/jclp.23402
